# The Association of Sociodemographic Variables and Unhealthy Behaviors With Limitations in Activities of Daily Living Among Thai Older Adults: Cross-sectional Study and Projected Trends Over the Next 20 Years

**DOI:** 10.2196/42205

**Published:** 2023-06-06

**Authors:** Jiraluck Nontarak, Kanitta Bundhamcharoen, Orawan Prasitsiriphon, Wichai Aekplakorn

**Affiliations:** 1 Department of Epidemiology Faculty of Public Health Mahidol University Bangkok Thailand; 2 International Health Policy Program Ministry of Public Health Nonthaburi Thailand; 3 College of Population Studies Chulalongkorn University Bangkok Thailand; 4 Department of Community Medicine Ramathibodi Hospital Faculty of Medicine, Mahidol University Bangkok Thailand

**Keywords:** ADL limitation, National Health Examination Survey, older adults, activities of daily living, physical activity, disability

## Abstract

**Background:**

Extended life spans have led to an increase in the number of older people and an increase in the prevalence of disability among people older than 60 years of age.

**Objective:**

This study aims to investigate the association of sociodemographic variables and unhealthy behaviors with limitations in activities of daily living (ADL) among Thai older adults. The study also projects the number of older individuals likely to experience ADL limitations in the next 20 years.

**Methods:**

We performed sex-specific multinomial logistic regression analysis based on the 5th Thai National Health Examination Survey in 2014 to investigate the association between sociodemographic variables and health behaviors with ADL limitations among Thai older adults. Age- and sex-specific prevalence estimates of ADL limitations were obtained by applying the same models. These estimates were combined with population projections up to 2040 from the Office of the National Economic and Social Development Board, Thailand, to generate projections of older individuals with ADL limitations.

**Results:**

Age and physical activity were significant factors for both sexes, with age positively associated with the level of ADL limitations and low physical activity associated with an increased relative probability of mild or moderate to severe ADL limitations compared to individuals with no ADL limitation (1.2-2.2 times). Other variables such as education, marital status, diabetes, hypertension, smoking, alcohol consumption, and having a fruit- and vegetable-based diet showed significant associations, but the results varied regarding sex and levels of ADL limitations. This study also projected the number of older adults with mild and moderate to severe ADL limitations over the next 20 years from 2020 to 2040, revealing an increase of 3.2 and 3.1 times, respectively, along with a significant increase in men compared to that in women.

**Conclusions:**

This study identified age and physical activity as significant factors associated with ADL limitations in older adults, while other factors showed varying associations. Over the next 2 decades, projections suggest a significant increase in the number of older adults with ADL limitations, particularly men. Our findings emphasize the importance of interventions to reduce ADL limitations, and health care providers should consider various factors impacting them.

## Introduction

An aging society is characterized by a state where individuals aged 60 years and older constitute over 10% of the total population [1]. By 2022, Thailand will have a complete aging society, and by 2030, it may become a superaged society. In just 9 years, Thailand is projected to become a hyperaged society, with an aging population approaching 20% of the nationwide population [2]. The increasing number of older people with longer life expectancies raises questions about whether they live longer lives with good health and how unhealthy behaviors affect their future lives. Furthermore, longer life expectancies and the concomitant increase in the incidence of noncommunicable diseases, such as hypertension, diabetes, stroke, and coronary artery disease, have led to questions about the well-being of older individuals [3,4]. These healthy states lead to a disabled state and to complete dependency throughout life. The dependence of older individuals is one of the major challenges faced by the Thai health care system. The National Health Examination Survey (NHES) also found that the prevalence of at least one functional limitation in activities of daily living (ADL) slightly increased from 11.1% in 2015 to 11.4% in 2019 [5]. The prevalence of at least one limitation increased with age, accounting for 11.1% in those aged 60 years to 17.6% among those aged 80 years [5].

Unhealthy behaviors such as low physical activity and having a high BMI, high cholesterol, and high blood sugar are known to increase the likelihood of disability among older adults [6-8]. Handgrip strength and a usual walking speed are objective measures of muscle strength and physical function and are useful in identifying people at risk of functional limitations, particularly ADL disabilities [9,10]. Physical activity is one of the most significant parameters for predicting functional limitations and is associated with physical performance [11]. Encouraging policy makers to promote a healthy lifestyle among older people could help reduce the incidence of functional limitations in this population.

Governments require precise and prompt demographic information that is categorized by age, gender, and other pertinent factors to prepare for demographic changes and effectively implement policies and initiatives to address the effects and possibilities of an aging population. The United Nations suggests that governments be devoted to gathering, sharing, and evaluating demographic statistics, such as those pertaining to aging populations and older individuals. In Thailand, the studies on the correlation of social determinants of health and unhealthy behaviors with ADL limitations among older people are limited. Moreover, predictive values of personal health behavior and social determinants of health have seldom been applied to project the volume of older individuals with different ADL limitation levels. These estimates are crucial for driving health policy and addressing interventions that could decrease the number of disabilities among older individuals in the future [12,13]. Therefore, this study aimed to investigate the association of sociodemographic variables and unhealthy behaviors with ADL limitations among older people in Thailand and project the number of individuals likely to experience ADL limitations in the next 20 years (2020-2040).

## Methods

### Data and Management

Our study was based on two data sources: (1) the Thai NHES in 2014, and (2) population projections by 5-year age group and sex in Thailand.

#### The Thai NHES

Data from the Thai NHES in 2014 were obtained [14]. This survey constitutes a large cross-sectional study using stratified 4-stage sampling to provide nationally and regionally representative samples of the Thai population, conducted every 5 years. The sampling method has been described elsewhere [14,15]. Briefly, stratified random sampling was applied. The first stage of sampling was systematic selection from 5 provinces in each of the 4 regions, including Bangkok. In total, 3-5 districts were randomly selected for each chosen province. Next, 13 to 14 electoral units in municipality areas or villages in a municipality area for each district were randomly selected. Finally, individuals aged ≥1 year by selected electoral units and villages, sex, and age group were randomly chosen. A total of 19,468 participants were sampled in 2014. This study recruited 7366 individuals aged 60 years and older. Demographic information of the participants was collected using face-to-face interviews, physical examinations, and laboratory tests with assured data quality and data management.

#### Dependent Variable

ADL limitations were assessed using the Barthel index [16]—an ordinal scale used to measure performance in ADL. ADL limitations were defined as the inability to perform basic self-care activities without assistance, including feeding, dressing, bathing, using the toilet, and transferring from beds or chairs. Each item was rated on a 3-item ordinal rating scale (0=unable, 1=needs help, and 2=independent). The final score was obtained by summing up the scores of all items, resulting in a 100-point score, in accordance with the guidelines for interpreting Barthel scores of Shah et al [17]. ADL scores were categorized into 3 levels: moderate to severe limitations (0 to 90 points), mild limitations (91 to 99 points), and no limitation (100 points).

#### Health Status Variables

Hypertension was defined as a diagnosis based on a systolic blood pressure of ≥140 mm Hg, diastolic blood pressure of ≥90 mm Hg from physical examinations, or self-reports of using antihypertensive medication.

Diabetes mellitus was defined as a diagnosis based on a fasting plasma glucose level of ≥126 mg/dL from laboratory blood testing or self-reports of diagnosis from physicians, and currently receiving medical treatment.

BMI was categorized as follows: underweight (<18.5 kg/m²), normal weight (18.5≤BMI<25 kg/m²), and overweight (≥25 kg/m²).

Smoking status was categorized as follows: never smoked, current smokers (people who have smoked in the past 12 months), and former smokers (people who have smoked previously and not in the past 12 months).

Alcohol consumption was defined as consuming at least 1 alcoholic drink (12.5 g/day) in the past 12 months.

Physical activity level was categorized as high (≥150 minutes/week of combined intensity) and low (<150 minutes/week of combined intensity) in accordance with the global physical activity questionnaire of the World Health Organization [18].

Fruit and vegetable consumption was categorized as high (≥5 portions/day) and low (<5 portions/day) [19].

#### Population Projection

To project the number of older adults with ADL limitations in the future, we required population projections by age and sex. We used population projections from the National Economic and Social Development Board, which were based on population registration data from 2010 to 2015, collected by the Ministry of Interior and were generated using the cohort-component method. We selected the medium fertility assumption, which assumes that the fertility rate will decline from 1.62 in 2010 to 1.30 in 2040. The mortality assumption was based on life expectancy, which is projected to increase from 70.5 years to 76.8 years for men and from 77.8 years to 83.2 years for women between 2010 and 2040. The mortality pattern was created using a relational logit model. Lastly, a migration rate of 0 was used in the population projections.

### Statistical Analyses

Descriptive statistics, including prevalence, proportion, mean, and SD, were used. All analyses were weighted to account for this, and statistical significance was set at *P*<.05. To predict prevalence, we first selected potential covariate variables associated with ADL limitations. The results showed that age, sex, residential area, education, employment status, behavior risks, and health status were associated with ADL limitations (details are included in Table S1 in Multimedia Appendix 1). All analyses in this study were performed using STATA (version 11; StataCorp).

Multinomial logistic regression was used to predict the prevalence of ADL limitations by sex while controlling for potential covariates. This type of model characterizes the probability of a participant's decision for a discrete choice [20]. Once the multinomial regression model was generated, estimated marginal standardization [21,22] was used to predict prevalence rates of ADL limitations among Thai older individuals based on fixed values of potential covariates. The prevalence rates of ADL limitations were presented by age group (60-64, 65-69, 70-74, 75-79, and 80 years and older) and sex (female and male).

Our projection of older adults with ADL limitations from 2020 to 2040 involved a deterministic model. This type of model does not include elements of randomness, so the same initial conditions will present the same results of the output model. The estimated age-specific prevalence rates of ADL limitations were multiplied by the population projection described in the data section and the rate of change in the prevalence of ADL limitations. This allowed us to assess the effect of changes in the demographic structure of the older population on the total number of people with ADL limitations. Direct age standardization was applied to calculate the prevalence rate of ADL limitations in 2014 and 2019. Based on this, we assumed that the prevalence rate of ADL limitations increased by 23% in men and 8% in women every 5 years. The applied standard population constituted the population structure in 2014 by sex, using sample weights.

### Ethical Considerations

This study was approved by the Human Research Ethics Committee of the Faculty of Public Health, Mahidol University (REC.MUPH 96/2022). The NHES study was approved by the Ethical Review Committee for Research in Human Subjects, Ministry of Public Health, and all participants provided written informed consent.

## Results

### Sample Characteristics

Overall, among 7306 respondents, the mean age in 2015 was 69.7 (SD 7.6) years, and the highest proportion of individuals (n=2493) were 34.1% in the age group of 60-64 years. Nearly equal proportions of men and women were observed (44.1% vs 55.9%, respectively). The prevalence rates of ADL limitations were 9.2% (n=672) for the mild level and 19.3% (n=1410) for the moderate to severe level.

As shown in Table 1, Thai older men and women without ADL limitations were generally younger than those with mild and moderate to severe ADL limitations. In total, 3343 of 5224 (64%) Thai older men and women without ADL limitations, 336 of 672 (50%) individuals with mild ADL limitations, and 635 of 1410 (45%) individuals with moderate to severe ADL limitations were aged between 60 and 69 years. Women comprised 52.6% (2748/5224) of Thai older adults without ADL limitations, while 62.3% (419/672) and 65.1% (918/1410) of those with mild and moderate to severe ADL limitations, respectively, were female. Older adults without and those with mild ADL limitations were more likely to live in urban areas (2737/5224, 52.4% and 368/672, 54.8%) than those with moderate to severe ADL limitations (682/1410, 48.4%).

Furthermore, the proportion of individuals with no education attainment increased with the level of ADL limitation (no limitation: 4378/5224, 83.8%; mild limitation: 587/672, 87.3%; moderate to severe limitation: 1282/1410, 90.9%). The proportion of individuals with an abnormal BMI increased with the level of ADL limitation (no limitation: 2304/5224, 44.1%; mild limitation: 317/672, 47.2%; moderate to severe limitation: 681/1410, 48.3%), particularly among underweight individuals. Additionally, one-third of older adults without ADL limitations were single, while the proportion of single individuals among those with ADL limitations was higher (mild limitations: 291/672, 43.3%; moderate to severe limitations: 575/1410, 40.8%).

**Table 1:** Description of sample characteristics by level of limitations in activities of daily living (ADL; N=7306).

Characteristics		No ADL limitation (n=5224), n (%)	Mild ADL limitations (n=672), n (%)	Moderate to severe ADL limitations (n=1410), n (%)
**Age group (years)**				
	60-64	1985 (38.0)	180 (26.8)	328 (23.2)
	65-69	1358 (26.0)	156 (23.2)	307 (21.8)
	70-74	904 (17.3)	140 (20.8)	276 (19.6)
	75-79	575 (11.0)	98 (14.6)	264 (18.7)
	≥80	402 (7.7)	98 (14.6)	235 (16.7)
**Sex**				
	Female	2748 (52.6)	419 (62.3)	918 (65.1)
	Male	2476 (47.4)	253 (37.7)	492 (34.9)
**Residential area**				
	Urban	2737 (52.4)	368 (54.8)	682 (48.4)
	Rural	2487 (47.6)	304 (45.2)	728 (51.6)
**Education**				
	No education	4378 (83.8)	587 (87.3)	1282 (90.9)
	Having an education	846 (16.2)	85 (12.7)	128 (9.1)
**Marital status**				
	Single	1740 (33.3)	291 (43.3)	575 (40.8)
	Married	3484 (66.7)	381 (56.7)	835 (59.2)
**BMI**				
	Normal	2920 (55.9)	355 (52.9)	729 (51.7)
	Underweight	319 (6.1)	46 (6.8)	130 (9.2)
	Overweight	1985 (38.0)	271 (40.4)	551 (39.1)
Having diabetes mellitus		946 (18.1)	159 (23.7)	278 (19.7)
Having hypertension		2837 (54.3)	423 (62.9)	888 (63.0)
**Smoking**				
	Never smoked	3380 (64.7)	461 (68.6)	1007 (71.4)
	Former smoker	1013 (19.4)	130 (19.3)	238 (16.9)
	Current smoker	831 (15.9)	81 (12.1)	165 (11.7)
**Alcohol consumption**				
	Nondrinker	4414 (84.5)	608 (90.5)	1270 (90.1)
	Drinker	810 (15.5)	64 (9.5)	140 (9.9)
**Fruit and vegetable intake**				
	<5 portions	1306 (25.0)	173 (25.8)	275 (19.5)
	>5 portions	3918 (75.0)	499 (74.2)	1135 (80.5)
**Physical activity level**				
	Low	1097 (21.0)	214 (31.9)	578 (41.0)
	High	4127 (79.0)	458 (68.1)	832 (59.0)

The prevalence of hypertension tended to increase with the level of ADL limitation, but that of diabetes mellitus did not, the latter having been the highest in older adults with mild ADL limitations (159/672, 23.7%). The proportion of individuals with prior and current smoking experience decreased with an increase in the level of ADL limitation (no limitation: 1844/5224, 35.5%; mild limitation: 211/672, 31.4%; moderate to severe limitation: 403/1410, 28.6%). The proportion of alcohol drinkers was higher among individuals with no ADL limitation (810/5224, 15.5%) than among those with mild limitations (64/672, 9.5%) and those with moderate to severe limitations (140/1410, 9.9%). Furthermore, the proportion of individuals with insufficient fruit and vegetable consumption was higher among those with mild ADL limitations (173/672, 25.8%) than in those with no limitation (1306/5224, 25%) and those with moderate to severe limitations (275/1410, 19.5%), while that of individuals with low physical activity levels increased with the level of ADL limitations (no limitation: 1097/5224, 21%; mild limitations: 214/672, 31.9%; moderate to severe limitations: 578/1410, -41%).

### Association Between ADL Limitation Level Across Sociodemographic Variables and Health Status

Table 2 shows the adjusted odds ratios for the association between ADL limitation level and various sociodemographic variables. The baseline group had no ADL limitation. After adjusting for other variables, we observed that among women, the associations between mild limitations and sociodemographic variables were significant among older adults (ie, those aged ≥70 years), those with an education, those with diabetes mellitus, those who drink alcohol, and those with low physical activity levels. Among men, the adjusted odds ratio for the association between mild limitations and sociodemographic variables was significant with respect to age (ie, ≥70 years), having hypertension, current smoking, current drinking alcohol, having inadequate fruit- and vegetable-based diets, having low physical activity levels, and having a BMI in the overweight range.

Moderate to severe ADL limitations were significantly associated with all age groups among women and significantly associated with age groups of ≥70 years among men. Having hypertension had a significant association with moderate to severe ADL limitations among men but not among women. Having low physical activity levels exhibited a stronger significant association with having moderate to severe ADL limitations than with having high physical activity levels. Furthermore, the association between moderate to severe limitations and having a BMI in the underweight range was significant in both men and women.

**Table 2:** Multinomial logistic regression for levels of limitation in activities of daily living (ADL) and sociodemographic variables.

			Mild ADL limitations										Moderate to severe ADL limitations							
			Women					Men					Women					Men		
			AOR^a^		*P* values		AOR			*P* values		AOR			*P* values		AOR			*P* values
**Age group (years; reference: 60-64 years)**																				
	65-69	1.250		.10		1.018			.91		1.462			<.001		1.007			.95	
	70-74	1.642		.003		1.704			.001		2.110			<.001		1.319			.03	
	75-79	1.894		<.001		1.565			.009		2.914			<.001		1.788			<.001	
	≥80	2.021		<.001		3.497			<.001		3.154			<.001		1.593			.001	
**Residential area (reference: urban)**																				
	Rural	0.933		.33		0.905			.36		1.378			<.001		0.964			.62	
**Education level (reference: no education)**																				
	Having education	0.634		.001		1.009			.94		0.496			<.001		0.700			<.001	
**Marital status (reference: single)**																				
	Married	0.648		<.001		1.278			.07		0.949			.40		1.650			<.001	
Having diabetes mellitus			1.363		.02		1.254			.08		1.185			.02		0.934			.52
Having hypertension			1.015		.86		1.38			.006		0.998			.98		1.777			<.001
**BMI category (reference: normal)**																				
	Underweight (BMI<18.5)	1.35		.06		0.916			.70		1.401			.01		1.683			.005	
	Overweight (BMI≥25)	0.98		.83		1.49			<.001		1.295			<.001		0.947			.58	
**Smoking status (reference: not smoking)**																				
	Former smoking	0.954		.76		1.527			.002		1.007			.96		1.099			.27	
	Current smoking	0.678		.12		1.546			.001		1.070			.57		1.047			.67	
Drinkers			0.679		.04		0.704			.004		0.682			.03		1.001			.99
Insufficient fruit- and vegetable-based diet			1.016		.86		1.704			<.001		0.676			.001		1.078			.41
Low physical activity			1.223		.02		2.173			<.001		1.814			<.001		1.571			<.001

^a^AOR: adjusted odd ratio.

### Starting Population and Probability Prediction of the Prevalence of ADL Limitations by Level

**Figure 1:**
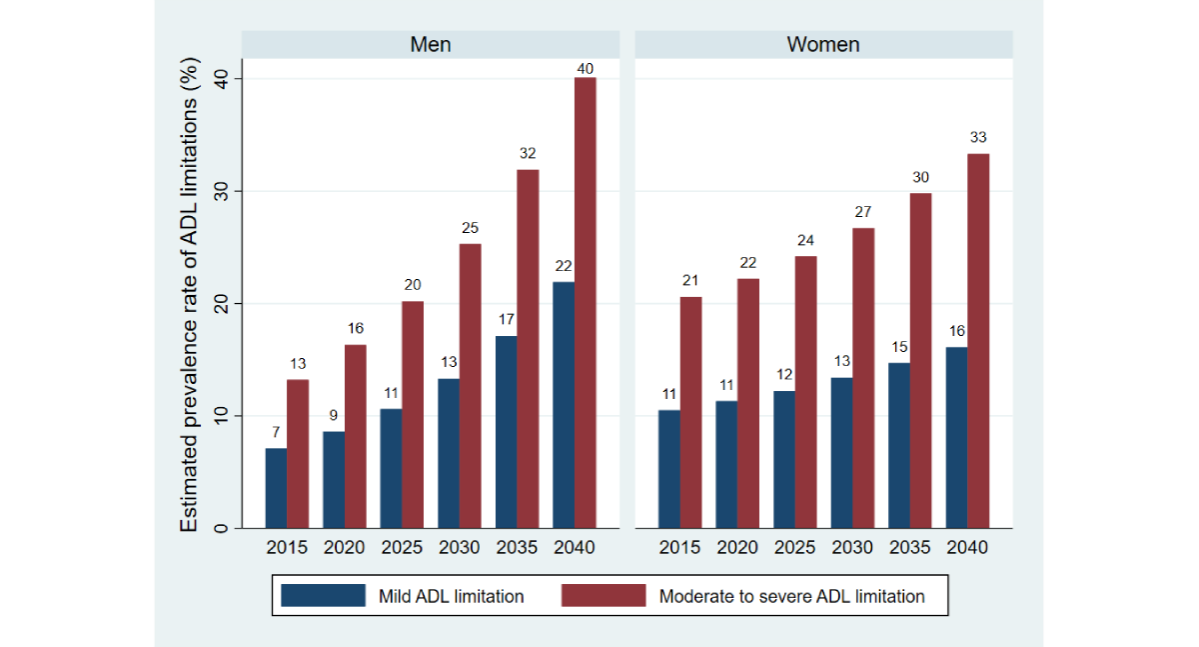
Estimated prevalence rate of ADL limitations among older individuals by level of ADL limitation and gender in Thailand in 2015-2040. ADL: activities of daily living.

The overall population volume in 2015 was 4,304,000 for older men and 5,676,000 for older women. After adjusting for other variables in the model, the probability prediction of prevalence from postestimation regression was divided by age group and sex. The prevalence of mild functional ADL limitations was 7.1% (n=306,225) among older men and 10.5% (n=593,333) among older women, and for moderate to severe ADL limitations, it was 13.2% (n=569,730) among men and 20.6% (n=1,171,028) among women at the base year. The prevalence rates of functional ADL limitations increased by 23% among men and 8% among women every 5 years, in accordance with the rate of change in the prevalence of ADL limitations from the NHES from 2014 to 2019. Figure 1 presents the estimated prevalence rate of ADL limitations among older individuals by level of ADL limitation and gender.

### Estimated Number of Older Individuals by the Level of ADL Limitations

**Figure 2:**
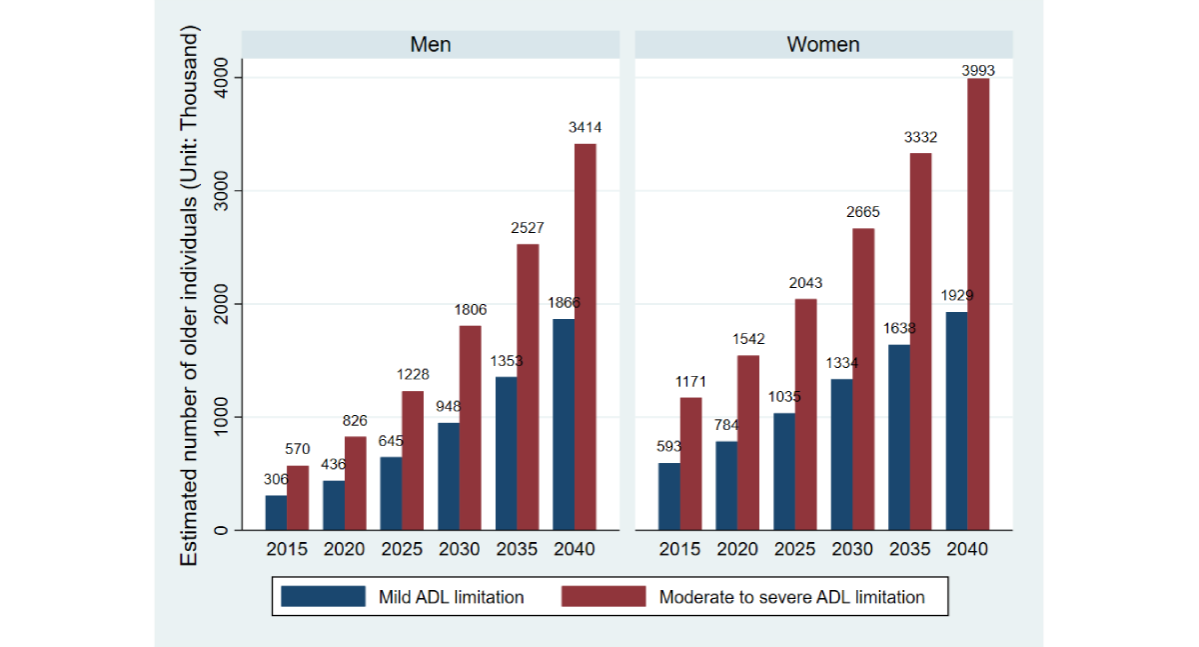
Estimated number of older Individuals by the level of ADL limitations in Thailand in 2015-2040. ADL: activities of daily living.

Over 20 years, an increase in the predicted number of older individuals with ADL limitations was more likely among women than among men (20.3 million vs 15.1 million, respectively). Among men, the predicted number of people with mild ADL limitations in the population increased from 436,000 in 2020 to 1,866,000 in 2040. The number in the moderate to severe group also increased, from 826,000 to 3,414,000. Among women, the number of older people with mild ADL limitations showed a linear increasing pattern from 784,000 in 2020 to 2,040,000 in 2040, while that in the moderate to severe ADL limitations group increased from 1,542,000 to 3,993,000. The population estimates for both groups are presented in Figure 2.

## Discussion

### Main Findings, Interpretations, and Comparisons With Related Studies

This study used data from a nationwide health survey among older individuals to investigate the association of sociodemographic variables and unhealthy behaviors with ADL limitations, and to estimate the predicted number of people living with ADL limitations from this association. The study identified age and physical activity as factors significantly associated with ADL limitations in older adults, while other factors showed varying associations. Over the next 2 decades, our projections suggest a significant increase in the number of older adults with ADL limitations, particularly men.

This study found that ADL limitations remarkably increased with age among men and women. In addition, the estimated number of Thai older people with ADL limitations continuously increased over 20 years, and women accounted for a higher number of older people with ADL limitations than men. Consistent with several studies, our study found a significant association between ADL limitations and sociodemographic factors such as age, having chronic health conditions, and having unhealthy behaviors. It confirmed the profound effect of increasing age and chronic diseases on leading ADL limitations in older individuals [6,9,10,12,23].

Our findings indicate that the prevalence of ADL limitations increased with age. Similarly, a previous study [24] reported that the prevalence of disability increased with older age. Older people presented a higher prevalence of functional limitations than younger people. Regarding chronic health conditions, the study revealed that having diabetes or hypertension was related to ADL limitations, which was consistent with previous studies. Older adults with major chronic illnesses exhibited higher prevalence rates of disability across all forms of ADL including bathing, dressing, walking, eating, using the toilet, and transferring in and out of bed [25,26]. Another longitudinal aging study in India found that among older individuals, having preexisting chronic conditions, such as hypertension, diabetes, psychiatric disorders, or stroke, was strongly associated with at least one ADL limitation [27]. Unhealthy behaviors also showed associations with disability among older adults. Similar to previous studies [23,28,29], an increased likelihood of incident disability is relevant to unhealthy lifestyles and health-related behaviors, and the risk increases with an increase in the number of unhealthy behaviors. Therefore, our predictive model strongly aligns with those of related studies.

Our study used estimated marginal standardization from predictions of a fitted model [21,22] at fixed values of potential covariates to forecast the number of ADL limitations among older Thai individuals. The model was controlled for any potential covariates, such as health conditions, low physical activity, insufficient fruit and vegetable intake, and sociodemographic status. The average number of ADL limitations was indicated by age, sex, and level of ADL limitations. The adjusted predictions refer to predictive values that are evaluated at fixed values for all covariates influencing functional limitations. Thus, this study endeavors to fill the gaps of other studies encountering limitations in terms of demographic variables such as health conditions, educational background, and unhealthy behaviors, among others [30]. The assumption of our model was that the measurement of ADL limitations, associated health conditions and other sociodemographic variables, was taken only at baseline in 2014; changes over time were not accounted for, and the risk associated with ADL limitations was assumed to be constant over time. The proportion of changes in ADL limitations over the study period increased by 23% among men and 8% among women, which was estimated by the differential between the prevalence of ADL limitations in 2014 and 2019. This change might reflect the real situation of ADL limitations in Thailand.

Our estimation of the average number of ADL limitations per year was 752,530 among men and 1,014,710 among women. Women were approximately 1-fold less likely to live with any ADL limitation than men. One explanation is that men exhibited a poorer health status and lifestyle and were more likely to be current alcohol consumers and smokers [31]. Furthermore, a study on the number of years lived with mobility limitations in older populations in Thailand found that women spent significantly more years with any limitation than men, and the number of years lived with severe limitations was notably constant across ages [32]. Our findings confirmed those of other studies that longer life spans do not necessarily indicate a good health status. At the age of 65 years, women were more likely to have a higher chance of experiencing mild ADL limitations (28.8%) and moderate to severe ADL limitations (46.5%) than those with no ADL limitation. However, our results do not provide supportive evidence of an association among men aged 65 years but rather indicate that increasing age is related to having any ADL limitation in both sexes. Regarding chronic diseases, older people with diabetes mellitus or hypertensive disorder were more likely to have any ADL limitation.

Overall, the number of older Thai individuals will gradually increase over the next 20 years. This study found that the number of moderate to severe ADL limitations would reach 7.4 million in 2040, which is approximately 3-fold the current volume of individuals with moderate to severe ADL limitations. Our results project a larger number of individuals with mild ADL limitations than that suggested by Tantirat et al [30], who estimated that the number of individuals needing assistance would total to 318,980. However, the difference might be due to the various methods used in the studies, including the definition of ADL limitation levels. Accordingly, a comparison of the number of older adults by ADL limitation level in this and other studies must be considered with caution because of these different definitions. In addition, the predicted number of older adults is sensitive to different calculation methods. However, the trends in limitations with other studies could be compared when using relatively similar definitions.

Our findings suggest improvements in creating a friendly environment for older individuals, such as improving sidewalks and stairs to prevent falls, along with an increased number of health promotion and prevention programs to enhance overall physical activity, prevent chronic diseases and other limitations, and improve the quality of life of older Thai individuals. Longer healthy life expectancy would also reduce health care expenditure in older populations.

### Study Strengths and Limitations

The strength of this study lies in the use of a large cross-sectional national health survey that represents the general nationwide population. Furthermore, potential confounding factors were considered in the analyses. However, this study has limitations, as encountered in cross-sectional studies. First, this type of epidemiological study cannot establish causal relationships. Moreover, the direction of causality is indeterminate; ADL limitations and the presence of chronic disease could be reciprocally related to each other. Second, older individuals with severe disabilities or bedridden conditions might not have been included in the survey due to the data collection process, where respondents were invited to health care centers. Therefore, the prevalence of ADL limitations from these surveys might have been underestimated.

In terms of methodology, this study encountered several limitations. We used a multinomial regression model to estimate the predicted probability of our outcome of interest. While certain demographic variables, such as marital status, residential area, and educational background, were considered in this model, it comprised a deterministic model that might not reflect the real situation [33]. Further studies should carry out multistage modeling to estimate the actual health state of older individuals in Thailand. Furthermore, additional empirical research on health status in the population, in terms of a longitudinal study, should be conducted to estimate several modeling parameters such as transit probability.

The findings of this study highlight the vast number of older Thai individuals who will potentially experience ADL limitations in the next 20 years. This information could be used in policy making, particularly in health promotion planning. Consequently, relevant factors can be modified to reduce the number of people with disabilities in the future.

## Appendix

Multimedia Appendix 1 Results of univariate logistic regression for ADL limitation level and socio-demographic variables.

## Abbreviations

ADL: activities of daily living
NHES: National Health Examination Survey
