# Supplementary material for: The Association of Sociodemographic Variables and Unhealthy Behaviors With Limitations in Activities of Daily Living Among Thai Older Adults: Cross-sectional Study and Projected Trends Over the Next 20 Years
Source: Asian Pac Isl Nurs J. 2023 Jun 6;7:e42205. doi: 10.2196/42205 (PMC10282915; doi:10.2196/42205)
Supplement: Multimedia Appendix 1 [file apinj_v7i1e42205_app1.docx]

# Multimedia Appendix 1

Results of univariate logistic regression for ADL limitation level and socio-demographic variables.

|  | **Mild Limitation** | | **Moderate to severe** | |
| --- | --- | --- | --- | --- |
| **ADL limitation** | **Unadjusted OR** | **P-value** | **Unadjusted OR** | **P-value** |
| **Age group (Ref. group = 60-64 years)** |  |  |  |  |
| 65-69 | 1.273 | .021 | 1.366 | *P*<.001 |
| 70-74 | 1.711 | *P*<.001 | 1.844 | *P*<.001 |
| 75-79 | 1.871 | *P*<.001 | 2.763 | *P*<.001 |
| 80+ | 2.696 | *P*<.001 | 3.532 | *P*<.001 |
|  |  |  |  |  |
| **Sex (Ref. group = Female)** |  |  |  |  |
| Male | 0.678 | *P*<.001 | 0.594 | *P*<.001 |
|  |  |  |  |  |
| **Living area (Ref. group = Urban)** |  |  |  |  |
| Rural | 0.911 | .059 | 1.172 | .002 |
|  |  |  |  |  |
| **Education level (Ref. group = No education)** |  |  |  |  |
| Having education | 0.758 | .001 | 0.514 | *P*<.001 |
|  |  |  |  |  |
| **Marital status (Ref. group = single)** |  |  |  |  |
| Married | 0.646 | *P*<.001 | 0.721 | *P*<.001 |
| **Having diabetes mellites** | 1.381 | .003 | 1.151 | .008 |
| **Having hypertension** | 1.402 | *P*<.001 | 1.400 | *P*<.001 |
|  |  |  |  |  |
| **Smoking (Ref. group = non-smoking)** |  |  |  |  |
| Former smoking | 0.942 | .383 | 0.793 | *P*<.001 |
| Current smoking | 0.715 | .001 | 0.671 | *P*<.001 |
|  |  |  |  |  |
| **Alcohol drinking** | 0.574 | *P*<.001 | 0.599 | *P*<.001 |
| **Insufficient fruit/vegetable diet** | 1.045 | .56 | 0.728 | *P*<.001 |
| **Low physical activity** | 1.762 | *P*<.001 | 2.614 | *P*<.001 |
|  |  |  |  |  |
| **BMI categories (Ref. group=Normal)** |  |  |  |  |
| Underweight | 1.162 | .10 | 1.615 | *P*<.001 |
| Overweight | 1.127 | .062 | 1.117 | .045 |
